# Antidepressant-Like Properties of Intrastriatal Botulinum Neurotoxin-A Injection in a Unilateral 6-OHDA Rat Model of Parkinson’s Disease

**DOI:** 10.3390/toxins13070505

**Published:** 2021-07-20

**Authors:** Veronica Antipova, Carsten Holzmann, Alexander Hawlitschka, Martin Witt, Andreas Wree

**Affiliations:** 1 Institute of Anatomy, Rostock University Medical Center, D-18057 Rostock, Germany; alexander.hawlitschka@med.uni-rostock.de (A.H.); martin.witt@med.uni-rostock.de (M.W.); andreas.wree@med.uni-rostock.de (A.W.); 2 Gottfried Schatz Research Center for Cell Signaling, Metabolism and Aging, Macroscopic and Clinical Anatomy, Medical University of Graz, A-8010 Graz, Austria; 3 Institute of Medical Genetics, Rostock University Medical Center, D-18057 Rostock, Germany; carsten.holzmann@med.uni-rostock.de; 4 Centre of Transdisciplinary Neuroscience Rostock, D-18147 Rostock, Germany

**Keywords:** hemiparkinsonian rat, 6-OHDA, botulinum neurotoxin-A, depression, striatum, behavior, forced swim test, tail suspension test, open field test, elevated plus maze test, correlation analysis

## Abstract

Parkinson’s patients often suffer from depression and anxiety, for which there are no optimal treatments. Hemiparkinsonian (hemi-PD) rats were used to test whether intrastriatal Botulinum neurotoxin-A (BoNT-A) application could also have antidepressant-like properties in addition to the known improvement of motor performance. To quantify depression- and anxiety-like behavior, the forced swim test, tail suspension test, open field test, and elevated plus maze test were applied to hemi-PD rats injected with BoNT-A or vehicle. Furthermore, we correlated the results in the forced swim test, open field test, and elevated plus maze test with the rotational behavior induced by apomorphine and amphetamine. Hemi-PD rats did not show significant anxiety-like behavior as compared with Sham 6-OHDA- + Sham BoNT-A-injected as well as with non-injected rats. However, hemi-PD rats demonstrated increased depression-like behaviors compared with Sham- or non-injected rats; this was seen by increased struggling frequency and increased immobility frequency. Hemi-PD rats intrastriatally injected with BoNT-A exhibited reduced depression-like behavior compared with the respective vehicle-receiving hemi-PD animals. The significant effects of intrastriatally applied BoNT-A seen in the forced swim test are reminiscent of those found after various antidepressant drug therapies. Our data correspond with the efficacy of BoNT-A treatment of glabellar frown lines in treating patients with major depression and suggest that also intrastriatal injected BoNT-A may have some antidepressant-like effect on hemi-PD.

## 1. Introduction

Parkinson’s disease (PD) is the most common and complex age-related chronic neurodegenerative movement disorder [1,2,3,4], hallmarked by the progressive loss of about 50–70% of dopamine neurons in the substantia nigra pars compacta (SNpc) and the reduction of dopamine (DA) in the caudate-putamen (CPu, striatum) [5,6,7], associated with classical primary (akinesia, bradykinesia, tremor, rigidity, and postural instability) and secondary motor symptoms (e.g., gait disturbance, micrographia, precision grip impairment, and speech problems) [8,9,10,11]. However, non-motor symptoms are increasingly recognized as relevant in the disease-state, given the associated alterations in mood (depression and anxiety) and cognition [12,13,14]. Throughout PD progression, motor impairments are generally preceded by non-motor symptoms such as depression, anxiety, olfactory deficit, sleep behavior disorder, and constipation, sometimes by up to ten years [13,15,16,17,18,19,20]. Non-motor symptoms of PD are very often overlooked though they are increasingly being investigated and have a very crucial impact on the clinical care and patient’s quality of life [10,20,21,22,23,24].

Depression is one of the most common psychiatric and neurodegenerative disorders; every fifth individual suffers from a mood disorder in his or her lifetime [25,26,27,28,29]. The World Health Organization estimates that major depression is the fourth most important cause worldwide of loss in disability-adjusted life years [30]. Many studies have reported an increased prevalence of depression in patients before the clinical onset of PD [31,32,33,34,35,36,37,38]. At the same time, depression, anhedonia, and anxiety are one of the most prevalent and serious comorbidities in patients with PD [39,40,41,42,43,44]. Estimates of the prevalence of depression in PD patients vary from 2.7% to 90%, but most surveys have suggested 40–50% levels [45,46,47,48,49,50,51,52,53]. Depression in PD is associated with great cognitive impairment [48,54], more rapid disease progression [55], and increased disability [56]. Theories associated with the etiology of depressive symptoms in PD argue that depression in PD is complex, “reactive”, and secondary to the psychosocial stress of chronic disease, and may result from changed serotonin brain chemistry that is related to the central dopaminergic deficiency associated with PD motor symptoms [57,58,59,60]. Anxiety is also highly prevalent in PD populations, with 60% of PD patients reporting anxiety [13,21,61,62,63,64].

Treatment options for PD are limited, with most of the current approaches based on restoration of DA levels that are effective in reducing motor symptoms, but associated with significant side effects [65,66,67]. At the same time, despite the high prevalence of neuropsychiatric symptoms in PD patients, there are also limited treatments targeting neuropsychiatric symptoms of PD [13,26,51,68,69,70,71,72,73,74]. Depression in particular is a common non-motor feature of PD, which frequently remains unrecognized and untreated [75,76]. In one of the few studies that explored the effects of antidepressants on cognitive function in PD patients, Dobkin et al., found no treatment-related improvements in cognition associated with the administration of the paroxetine and nortriptyline despite successful antidepressant effects [51,69]. Functional neuroimaging research showed that dopaminergic medications alter regional brain response during cognitive task performance in PD patients, but these alterations did not correlate with test performance [69,77,78,79]. Other studies even mentioned that dopaminergic medications may enhance or impair cognitive function in PD depending upon the nature of the task and basal levels of DA [68,80,81]. Notably, Cools et al. [80] showed that cognitive inflexibility decreases on dopaminergic therapy while impulsivity increases. Hälbig et al. [68] reported that PD patients’ performance on emotion recognition and reaction time tasks was significantly worse while “on” versus “off” dopaminergic therapy. Blonder et al. [69] examined the neuropsychological effects of dopaminergic pharmacotherapy in Parkinsonian depression and compared cognitive function in 28 non-demented depressed and non-depressed PD patients at two time-points: following overnight withdrawal and after the usual morning regimen of dopaminergic medications. The study revealed a significant interaction between depression and medication status on a facial naming task and three measures of verbal memory. In all cases, depressed PD patients performed more poorly while on dopaminergic medication than while off. The opposite pattern was seen in the non-depressed Parkinson’s group. Therefore, the authors concluded that administration of dopaminergic medication to depressed PD patients might carry unintended risks. Other studies demonstrated that some antiparkinsonian drugs such as dopamine receptor agonists and MAO-B inhibitors like selegiline and rasagiline, when administered in the recommended dose range for the treatment of PD, exerted limited antidepressant efficacy in patients with PD [52,82,83,84,85,86,87,88,89]. Also, mirtazapine, atomoxetine, and—interestingly—bupropion, a norepinephrine-dopamine reuptake inhibitor antidepressant, are reported as not effective in treating depression in PD patients [82,85,90,91,92].

Anxiety disorders, particularly generalized anxiety, panic, and social phobia, occur as a comorbidity in up to 40% of PD patients, and this rate is higher than in normal or other disease comparison populations [93,94,95,96,97,98,99,100]. As with depression in PD, the optimal pharmacologic treatment for anxiety in these patients has not yet been established [50,98,101,102,103,104].

In PD, DA depletion leads to hyperactivity of cholinergic interneurons in the striatum [105,106,107,108]. Botulinum neurotoxin-A (BoNT-A) inhibits the release of acetylcholine (ACh) in the peripheral nervous system and is also thought to act as a local anticholinergic drug when injected intrastriatally, i.e., into the CPu in hemiparkinsonian (hemi-) PD rats. In hemi-PD rats, injection of 1 ng BoNT-A into the DA-depleted CPu significantly diminished apomorphine-induced rotational behavior for at least 3 months, the effect fading thereafter [109,110,111,112,113,114,115,116,117,118,119,120].

Since the neurobiology and etiology of treatment options of depression in PD are not fully understood, we tested here the effect of the injection of 1 ng BoNT-A into the DA-depleted CPu of hemi-PD rats on depression-like and anxiety-like behaviors. It is known that the cholinergic system, including the striatum, plays a central role not only in cognition but also in depression [121,122,123,124,125,126,127] and anxiety [128,129,130,131,132,133,134].

It remains unclear whether alterations in dorsal or ventral corticostriatal circuits are the primary site of basal ganglia functional alterations in depression, particularly in its early stages [135]. Studies have generally focused on characterizing abnormalities in the “affective” ventral corticostriatal loops supporting emotional processes [136,137,138,139,140,141]. These results were interpreted in accordance with the classical neuroanatomical scheme of topologically organized ventral (“affective”) versus dorsal (“cognitive”) corticostriatal circuits [142,143,144]. Especially young, depressed patients demonstrated blunted ventral caudate/nucleus accumbens and putamen activation in the context of reward-based learning together with predominantly increased activation of ventralmedial prefrontal, anterior cingulate, and orbitofrontal cortical regions, i.e., major components of the so-called “ventral-affective” corticostriatal circuit [140,145,146,147].

Evidence has emerged in support of primary functional connectivity alterations involving dorsal as opposed to ventral corticostriatal circuits in adults [148] and young [149] depressed patients. Furman and colleagues [148] reported decreased functional connectivity between the ventral striatum and subgenual anterior cingulate cortex in adult depressed patients but increased connectivity between the dorsal caudate and dorsolateral prefrontal cortex.

Gabbay et al. [149] showed that adolescents with depression manifested increased intrinsic functional connectivity (iFC) between all striatal regions bilaterally and the dorsomedial prefrontal cortex (dmPFC), as well as between the right ventral caudate and the anterior cingulate cortex (ACC). Major depressive disorder (MDD) severity was associated with iFC between the striatum and midline structures including the precuneus, posterior cingulate cortex, and dmPFC. Also, evidence from Kerestes et al., has implicated alterations in the functional connectivity of dorsal “cognitive” corticostriatal loops in depressions [135]. The authors found that, compared with controls, depressed patients showed increased connectivity between the dorsal caudate nucleus and ventrolateral prefrontal cortex bilaterally. Increased depression severity correlated with the magnitude of dorsal caudate connectivity with the right dorsolateral prefrontal cortex. There were no significant differences between groups (medication-free patients with moderate-to-severe MDD and healthy control participants) in connectivity of ventral striatal regions [135].

Up to now, the study of anxiety has primarily focused on the amygdala, bed nucleus of the stria terminalis (BNST), hippocampus (HPC), and prefrontal cortex (PFC) [150,151]. Actually, the dorsal striatum is implicated in automatic responses and habit formation, the ventral striatum may be more involved in anxiety, given its importance in emotional processes [152].

Several studies have shown the antidepressant efficacy of acetylcholinergic drugs [153,154,155,156] or dosage-dependent antidepressant effects mostly of scopolamine [157,158,159]. While studies suggest that some selective serotonin reuptake inhibitors (SSRI) like fluorexine or citalopram can improve depression in PD patients, others report that fluorexine has a lot of side effects and worsened the tremor and motor symptoms in PD patients [52,160,161,162,163,164,165,166,167,168].

In rodents, it has been shown that a 6-hydroxydopamine-(6-OHDA)-induced lesion in the medial forebrain bundle (MFB) is a suitable model to investigate depressive-like behavior and exploratory activity impairments associated with PD [169,170,171,172]. However, most studies on depression- and anxiety-like behavior in the 6-OHDA model were done on bilaterally lesioned animals of different strains of mice [173,174] and rats [101,170,175,176,177,178,179,180,181]. Interestingly, depression-like and anxiety-like behaviors in hemi-PD rats of the Wistar strain were rarely examined; mostly Sprague Dawley or Wistar Han rats were used [74,169,181,182,183], however, with contradictory results [74,182,184].

Depression-like behaviors have been demonstrated in rats with 6-OHDA lesions; however, the respective results have been inconsistent among studies [60,74,169,179,180,181,182,184,185,186]. In hemi-PD rats with complete or nearly complete unilateral 6-OHDA lesions of the MFB, several studies found increased immobility time in the forced swim test (FST) and decreased sucrose consumption, respectively [74,169,172]. In contrast, two studies using the same 6-OHDA lesion of the MFB reported that striatal DA depletion did not induce any change in depression-like behaviors as measured by the FST and/or sucrose preference [182,184]. It should be taken into account that methodological aspects such as the exact site of the 6-OHDA injection, the extent of DA depletion, modifications of methods of behavior tests, and time-point for the behavior tests could influence test results.

Likewise, the results of anxiety-like behaviors in different 6-OHDA rat models of PD are also inconsistent [172]. In rats with unilateral MFB lesions, several studies found that these rats showed increased anxiety-like behaviors in the elevated plus maze test (EPM), locomotor chamber, or social interaction tests [182,186]. Other studies using the same model, however, showed no effects on anxiety-like behaviors in the EPM, open field test (OFT), or acoustic startle response test [169,184,187]. The discrepancy could be also caused by the site of the 6-OHDA injection, the extent of the lesion, modification of anxiety paradigms, and time frame for the behavior tests. In addition, several studies also observed decreased or increased anxiety-like behaviors after bilateral 6-OHDA injection into the dorsal striatum [179,180].

In 1981, Dr. Jankovic initially injected BoNT into a patient for treatment of blepharospasm (BSP) [188] and subsequently published the results of the first double-blind, placebo-controlled trial of BoNT in cranial–cervical dystonia [189]. Subsequently, BoNT-A was approved in 1989 by the US Food and Drug Administration for the treatment of BSP and other facial spasms. BoNT-A was successfully evolved into a therapeutic modality for a variety of movements disorders including some motor symptoms in PD [190] such as dystonia [191,192,193,194,195,196,197], jaw tremors [198], limb rest tremor [198,199,200], freezing of gait [201,202,203,204], sialorrhea [197,205,206,207,208,209], overactive bladder [210,211,212,213], constipation [214,215], dyskinesias [192], captocormia [216,217,218], Pisa syndrome [219,220], dysphagia [221,222], apraxia of lid opening [223,224].

Therefore, in the present study, we aimed to examine a possible therapeutic effect of intrastriatal injections of BoNT-A on depression-like behavior in the hemi-PD rat model induced by MFB lesion, using the forced swim test and tail suspension test, and on anxiety-like behavior as well as a motor activity using the open field test and elevated plus maze test.

## 2. Results

### 2.1. Spontaneous Motor Tests

#### 2.1.1. Open Field Test

The OFT examines spontaneous motor and explorative activities as well as the anxiety-like behavior of rats [225,226,227].

**Figure 1:**
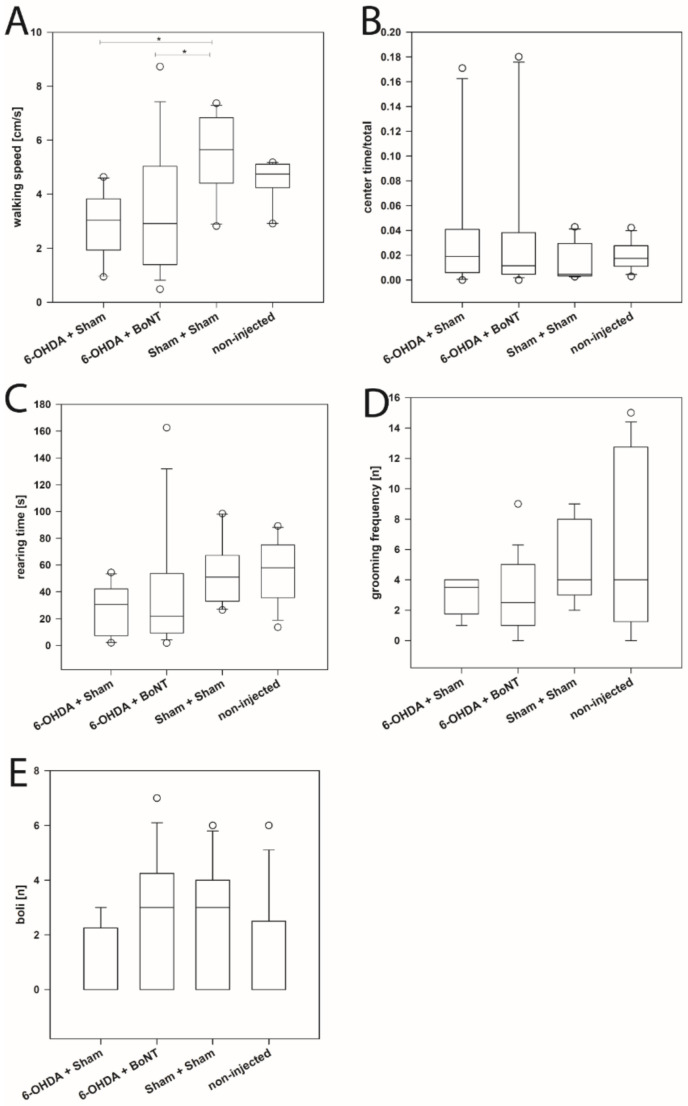
Open field test.(**A**) The walking speed of rats of the 6-OHDA + Sham and 6-OHDA + botulinum neurotoxin-A (BoNT) groups were significantly reduced in the OFT compared with the Sham + Sham group. (**B**) The center time/total time, (**C**) the rearing time, (**D**) the grooming frequency and (**E**) number of boli did not differ significantly between all four groups. Asterisks indicate significant differences after performing all multiple comparison procedures in pairs (Dunn’s method; * *p* < 0.05).

The walking speed during 10 min of the Sham + Sham (median: 5.65 cm/s) and non-injected groups (median: 4.75 cm/s) did not differ significantly (Figure 1A). In contrast, both the 6-OHDA + Sham (median: 3.03 cm/s) and the 6-OHDA + BoNT (median: 2.91 cm/s) groups showed significantly lower walking speed compared with the Sham + Sham group (*p* < 0.05) (Figure 1A). The further parameters related to curiosity and exploration activity (rearing time) or to anxiety (center time/total time, grooming frequency, number of boli) did not differ significantly between all four groups (*p* > 0.5) (Figure 1B–E).

#### 2.1.2. Elevated Plus Maze Test

**Figure 2:**
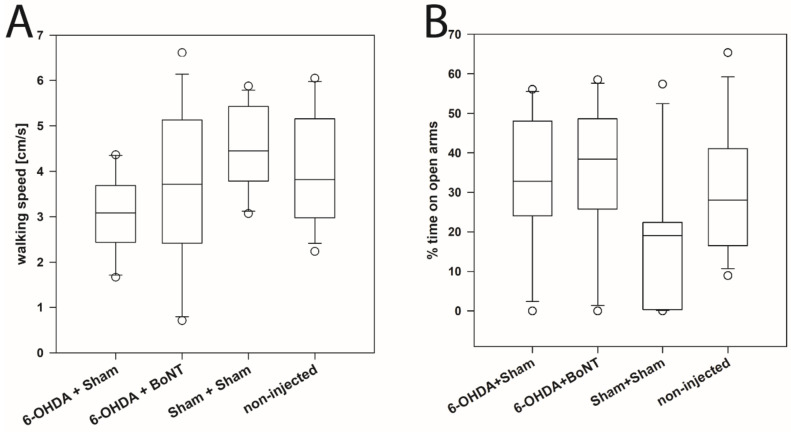
Elevated plus maze test. (**A**) The walking speed and (**B**) the % time spent on open arms of rats of the 6-OHDA + Sham, 6-OHDA + BoNT, Sham + Sham, and non-injected groups did not differ significantly.

This test further evaluates curiosity and anxiety-like behavior [228,229,230]. The walking speed during a 5-min time span in the four groups did not differ significantly: 6-OHDA + Sham (median: 3.08 cm/s), 6-OHDA + BoNT (median: 3.72 cm/s), Sham + Sham (median: 4.451 cm/s), non-injected (median: 3.82 cm/s) (Figure 2A). Also, the % of time spent on open arms did not differ significantly between the groups during the 5 min of observation time: 6-OHDA + Sham (median: 32.82%), 6-OHDA + BoNT (median: 38.44%), Sham + Sham (median: 19.04%), non-injected (median: 28.05%) (Figure 2B).

#### 2.1.3. Forced Swim Test

The FST is one of the most commonly used animal models for assessing depression-like behavior and also for the antidepressant efficiency of drugs [231,232,233].

**Figure 3:**
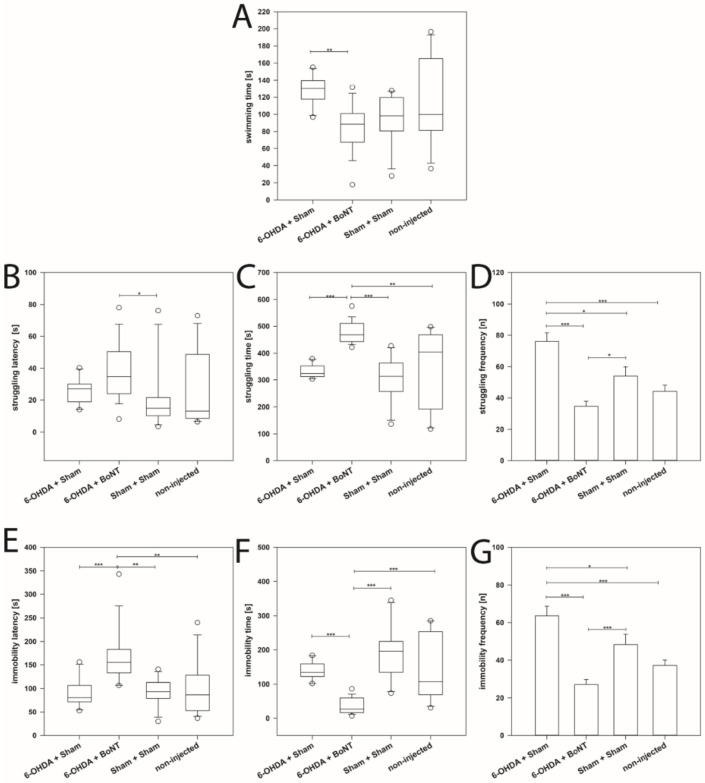
Forced swim test. (**A**) The swimming time of the 6-OHDA + BoNT-A group was significantly shorter than that of the 6-OHDA + Sham group. (**B**) The struggling latency in the 6-OHDA + BoNT group was significantly longer compared with the Sham + Sham group. (**C**) The struggling time of the 6-OHDA + BoNT group was significantly longer compared with all other groups. (**D**) The struggling frequency in the 6-OHDA + Sham group was significantly higher than in all other groups. (**E**) The immobility latency of the 6-OHDA + BoNT group was significantly longer compared with all other groups. (**F**) The immobility time of the 6-OHDA + BoNT group was significantly shorter compared with all other groups. (**G**) The immobility time of the 6-OHDA + BoNT group was significantly shorter compared with all other groups. The immobility frequency of the 6-OHDA + BoNT group was significantly lower than in the 6-OHDA + Sham group. Asterisks indicate significant differences after performing all multiple comparison procedures in pairs (Dunn’s method; * *p* < 0.05, ** *p* < 0.01, *** *p* < 0.001).

The mean swimming time of the 6-OHDA + BoNT group (median: 88.59 s) was significantly lower than that of the 6-OHDA + Sham group (median: 130.31 s) (*p* = 0.002) (Figure 3A). The struggling latency in the 6-OHDA + BoNT group (median: 34.68 s) was significantly increased compared with the Sham + Sham group (median: 14.88 s) (*p* < 0.05) (Figure 3B), while the struggling time of the 6-OHDA + BoNT group (median: 468.00 s) was significantly increased compared with all other groups (medians: 314.078 to 404.91 s) (*p* < 0.01) (Figure 3C). The struggling frequency in the 6-OHDA + Sham group was significantly higher (median: 76.10) than in all other groups: 6-OHDA + BoNT group (mean: 34.67), Sham + Sham group (mean: 54.00), non-injected group (mean: 44.17) (*p* < 0.05) (Figure 3D). Moreover, the struggling frequency in the 6-OHDA + BoNT group was significantly lower compared with the 6-OHDA + Sham group (*p* < 0.001) and the Sham + Sham group (*p* < 0.05) (Figure 3D).

As an important sign of the “lowered mood” and “despair” [234,235], the immobility latency of the 6-OHDA + BoNT group was significantly longer (median: 155.16 s) compared with all other groups: 6-OHDA + Sham group (median: 80.52 s, *p* < 0.001), Sham + Sham group (median: 93.20 s, *p* < 0.01), non-injected group (median: 86.42 s, *p* < 0.01) (Figure 3E). The immobility time of the 6-OHDA + BoNT group was significantly shorter (median: 26.883 s) compared with all other groups: 6-OHDA + Sham (median: 134.31 s), Sham + Sham group (median: 196.56 s), non-injected group (median: 107.35 s) (*p* < 0.001) (Figure 3F). Comparably, the immobility frequency in the 6-OHDA + Sham group was significantly higher (mean: 63.60) than in all other groups: 6-OHDA + BoNT group (mean: 27.06, *p* < 0.001), Sham + Sham group (mean: 48.36, *p* < 0.05), non-injected group (mean: 37.17, *p* > 0.001) (Figure 3G). The immobility frequency in the 6-OHDA + BoNT group was significantly lower compared with the 6-OHDA + Sham and the Sham + Sham groups (*p* < 0.001) (Figure 3G).

#### 2.1.4. Tail Suspension Test

The tail suspension test (TST) is an animal model commonly used for screening antidepressant drugs [236,237,238].

Rats in all four groups were scored as “0”. During the testing time of 60 s, none of the rats showed a pathological immobility time in the TST.

### 2.2. Correlating Open Field Test, Elevated Plus Maze Test, Forced Swim Test, and Apomorphine- and Amphetamine-Induced Rotations

Following OFT, EPM, and FST, all rats underwent apomorphine- and amphetamine-induced rotation tests about six weeks after vehicle or BoNT-A injection. With the correlation analysis, it is being tested whether or not there is a dependency of measured parameters in OFT, EPM, and FST on apomorphine- and amphetamine-induced rotations. If that were the case, the outcome of drug-induced rotations could possibly predict measures indicating depression-like or anxiety-like behavior in hemi-PD rats.

**Figure 4:**
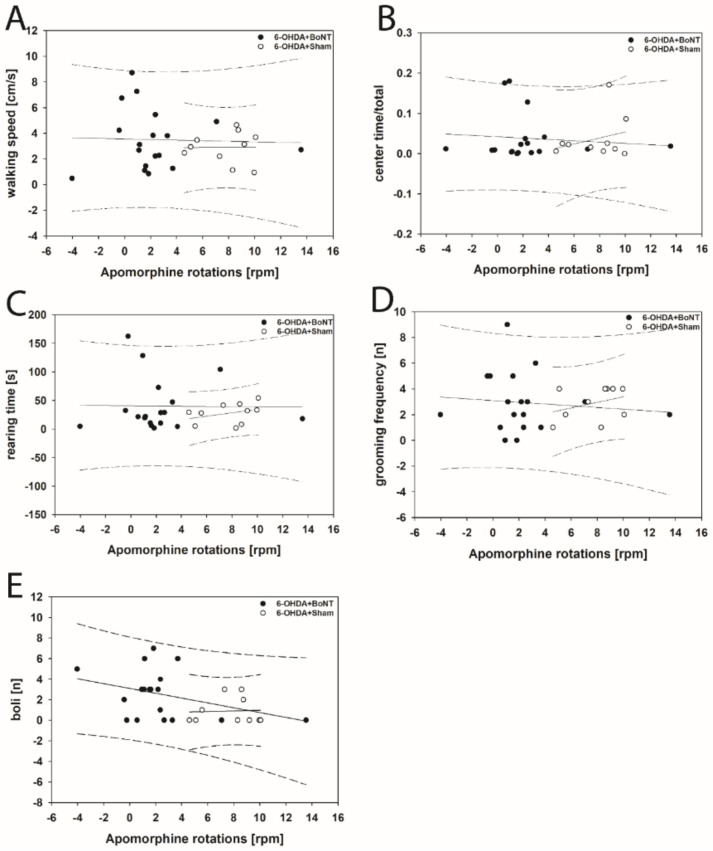
Scatter plots of parameters measured in the OFT and correlated with respective apomorphine-induced rotations of rats of the 6-OHDA + Sham and 6-OHDA + Botulinum neurotoxin-A (BoNT) groups. (**A**) Walking speed, (**B**) center time/total time, (**C**) rearing time, (**D**) grooming frequency, and (**E**) the number of boli did not correlate significantly in either group. Regression lines are displayed as solid lines and prediction intervals as dashed lines.

**Figure 5:**
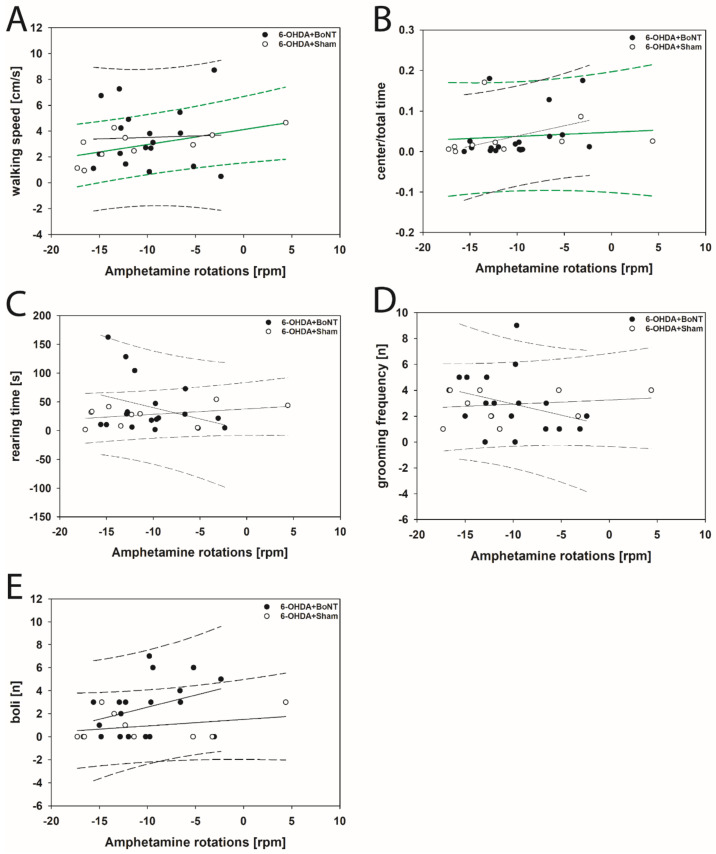
Scatter plots of parameters measured in the OFT and correlated with respective amphetamine-induced rotations of rats of the 6-OHDA + Sham and 6-OHDA + Botulinum neurotoxin-A (BoNT) groups. In 6-OHDA + Sham rats (**A**) walking speed and (**B**) center time/total time positively correlated with decreasing rotations. (**C**) rearing time, (**D**) grooming frequency, and (**E**) the number of boli did not correlate significantly in either group. Regression lines are displayed as solid lines and prediction intervals as dashed lines. Green-colored regression lines and prediction interval lines indicate significant differences (*p* < 0.05).

Neither parameter measured in the OFT significantly correlated with respective apomorphine-induced rotations of rats of the 6-OHDA + Sham or 6-OHDA + BoNT groups (Figure 4A–E). However, walking speed (rs = 0.648; *p* = 0.0377; Figure 5A) and center time/total time (rs = 0.661; *p* = 0.0332; Figure 5B) positively correlated with decreasing amphetamine-induced rotations in the 6-OHDA + Sham group. Rearing time (Figure 5C), grooming frequency (Figure 5D), and the number of boli (Figure 5E) did not correlate significantly with amphetamine-induced rotations in either group.

**Figure 6:**
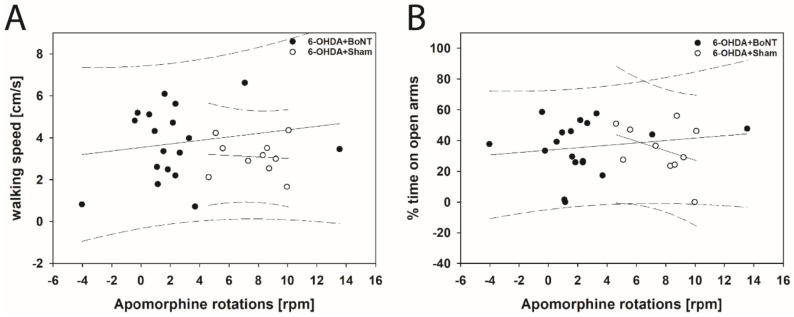
Scatter plots of parameters measured in the EPM and correlated with respective apomorphine-induced rotations of rats of the 6-OHDA + Sham and 6-OHDA + Botulinum neurotoxin-A (BoNT) groups. (**A**) Walking speed and (**B**) center time/total time did not correlate significantly in either group. Regression lines are displayed as solid lines and prediction intervals as dashed lines.

**Figure 7:**
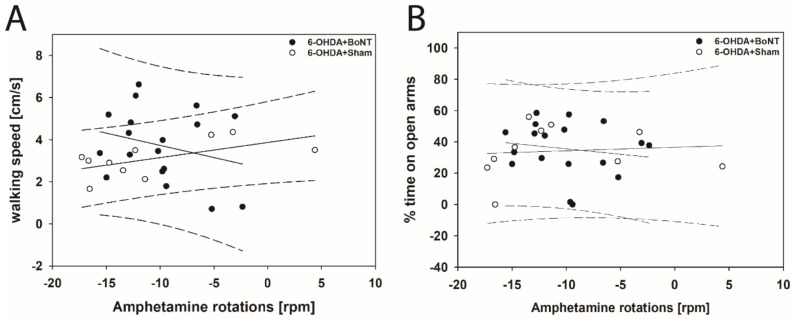
Scatter plots of parameters measured in the EPM and correlated with respective amphetamine-induced rotations of rats of the 6-OHDA + Sham and 6-OHDA + Botulinum neurotoxin-A (BoNT) groups. (**A**) Walking speed and (**B**) center time/total time did not correlate significantly in either group. Regression lines are displayed as solid lines and prediction intervals as dashed lines.

In EPM, neither walking speed nor center time/total time correlated significantly with the respective apomorphine- or amphetamine-induced rotations in rats of both the 6-OHDA + Sham and 6-OHDA + BoNT groups (Figure 6A,B and Figure 7A,B).

**Figure 8:**
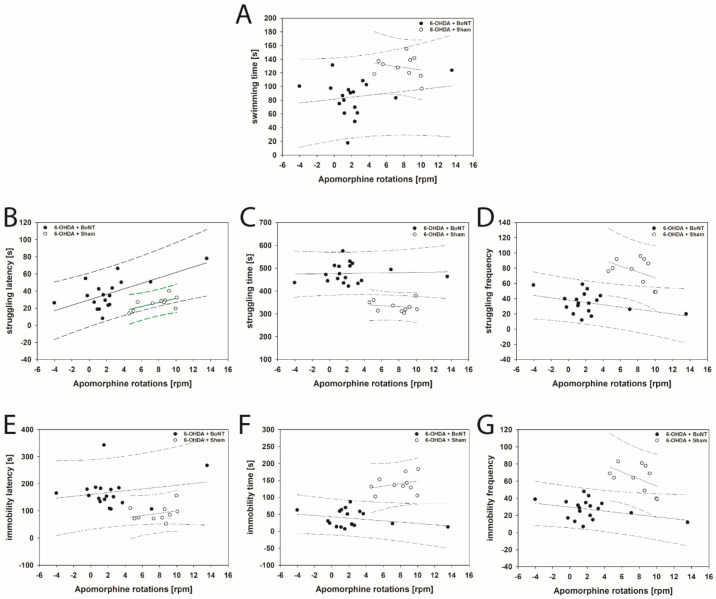
Scatter plots of parameters measured in the FST and correlated with respective apomorphine-induced rotations of rats of the 6-OHDA + Sham and 6-OHDA + Botulinum neurotoxin-A (BoNT) groups. In 6-OHDA + Sham rats (**B**) struggling latency correlated positively with increasing rotations. All other parameters in either group did not show significant correlations: (**A**) swimming time, (**C**) struggling time, (**D**) struggling frequency, (**E**) immobility latency, (**F**) immobility time, (**G**) immobility frequency. Regression lines are displayed as solid lines and prediction intervals as dashed lines. Green-colored regression lines and prediction interval lines indicate significant correlation (*p* < 0.05).

**Figure 9:**
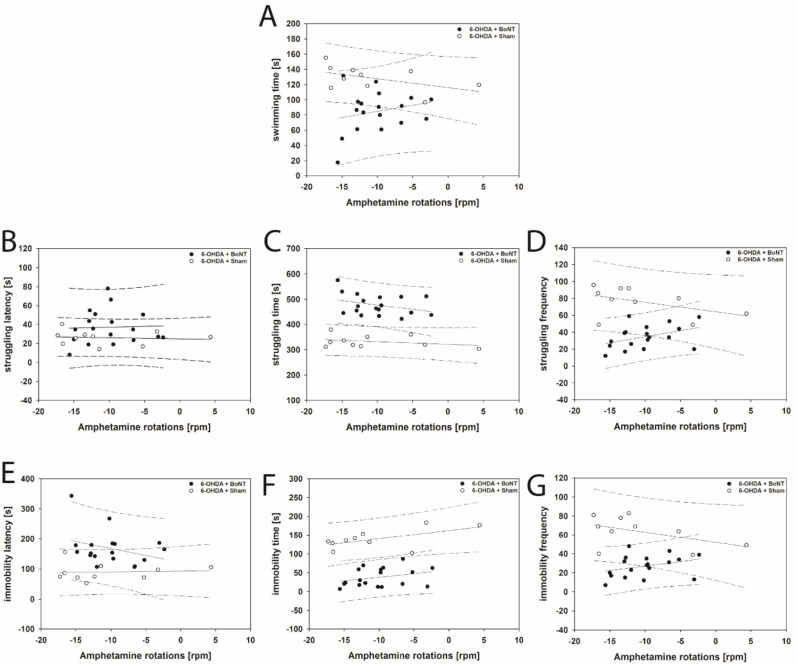
Scatter plots of parameters measured in the FST and correlated with respective amphetamine-induced rotations of rats of the 6-OHDA + Sham and 6-OHDA + Botulinum neurotoxin-A (BoNT) groups. All parameters in both groups did not show significant correlations: (**A**) swimming time, (**B**) struggling latency, (**C**) struggling time, (**D**) struggling frequency, (**E**) immobility latency, (**F**) immobility time, (**G**) immobility frequency. Regression lines are displayed as solid lines and prediction intervals as dashed lines.

The FST revealed that struggling latency in 6-OHDA + Sham rats correlated positively with increasing apomorphine-induced rotations (rs = 0.661; *p* = 0.0332; Figure 8B). In this parameter rats of the 6-OHDA + BoNT group just missed a significant positive correlation with increasing apomorphine-induced rotations (*p* = 0.054; Figure 8B). All other parameters measured in the FST in 6-OHDA + Sham and 6-OHDA + BoNT groups did not show significant correlations with apomorphine- and amphetamine-induced rotational behavior (Figure 8A,C–G and Figure 9A–G).

## 3. Discussion

The non-motor symptoms of PD are often present before diagnosis occurs in roughly 90% of patients; they dominate the clinical picture of advanced PD and contribute to severe disability, impaired quality of life, and shortened life expectancy [13,73,239,240]. Furthermore, in contrast to the dopaminergic symptoms of the disease, for which treatment is partially available, non-motor symptoms like depression and anxiety are common symptoms for which there are presently no optimal treatments [13,74,184,241,242,243,244].

The prevalence of anxiety disorders in PD ranges from 25 to 43%, and depression can affect up to 45% of patients with PD [245,246,247,248,249,250,251]. Depression is frequently the presenting symptom before significant motor symptoms are observed, and, therefore, may be considered a risk factor for PD and a prodromal symptom of PD [28,37,48,240,252].

Depression is therefore more a consequence of the disease process and not simply the result of psychological stress caused by the development of chronic disease. Anxiety is not simply a factor of motor impairment alone but reflects a neuropathological, disease-related susceptibility [86].

Typically used anti-depression medication includes tricyclic antidepressants (TCA), SSRI, serotonin and norepinephrine reuptake inhibitors (SNRI), monoamine-oxidase inhibitors (MAOI), and DA agonists [253]. Clinical trials have been conducted to investigate their therapeutic effect on depression in PD patients. Barone et al. [254] reported that treatment with rasagiline, an MAOI, did not improve symptoms of depression in PD patients. Atomoxetine, an SNRI, was reported to be not efficacious for depression in PD but might help to improve cognitive disorder and daytime sleepiness [255]. The DA agonist pramipexole was found to be able to improve symptoms of depression in patients with PD through a direct antidepressant effect [89]. A randomized clinical trial also established that the TCA nortriptyline was efficacious in the treatment of symptoms of depression, but not the SSRI paroxetine [255].

Selective serotonin reuptake inhibitors have the side effect of a worsening of resting tremors in many PD patients [13,255,256]. Moreover, it actually appears that the biochemical responsivity towards SSRI differs between PD patients with co-morbid depression and patients with depression alone [257]. It is therefore of clinical importance to find novel compounds for the treatment of depression and anxiety in PD [74].

The present experimental animal study is based on two crucial points: the respective results from the literature on the efficiency of anticholinergics on depression [153,154,155,156,157,158,159] including the results that BoNT-A is effective for the treatment of patients with major depression if injected into the brow muscles [258,259,260,261,262,263,264,265,266] and our own previous findings that bilaterally injected intrastriatal BoNT-A reduced anxiety in naïve Wistar rats [117]. To avoid unwanted side effects of systematically applied classical anticholinergic drugs, and hypothesizing that BoNT-A inhibits the release of ACh also as a local anticholinergic drug, we injected BoNT-A directly into the CPu in hemi-PD rats [109,110,112,113,114,116,117,118,120]. One ng of BoNT-A injected into the DA-depleted striatum in hemi-PD rats significantly annulled apomorphine-induced rotations for a minimum period of 3 months [109,110,112,113,114,116,117,118,120]. Thereafter, rotation behavior gradually increased again during the next 6 to 9 months [109,110,118,120].

Interestingly, the treatment of glabellar frown lines with the injection of botulinum toxin is one of the most prevalent procedures in esthetic medicine [185,267,268,269]. Recently, three randomized controlled trials have consistently shown that such effects can be used in the treatment of depression. Mostly female patients suffering from partly chronic and treatment-resistant unipolar depression experienced a quick, strong, and sustained improvement in depressive symptoms after a single glabellar treatment with BoNT-A as a sole or adjunctive therapy [258,259,260,262,266,267,270,271,272,273]. However, it is still obscure which neurochemical or neuroanatomical pathways this therapeutic approach with BoNT follows.

Taken together, we hypothesized that BoNT-A could also have antidepressant-like properties when injected intrastriatally into hemi-PD rats. Studying this, the behavioral effects of BoNT-A were examined in the FST and TST, measures of antidepressant-like activity. OFT and EPM both evaluated anxiety-like behavior. Unilateral injection of 6-OHDA into the MFB was used to produce distinct motor impairments detectable as DA agonist-induced asymmetric rotating behaviors [274,275,276]. However, this rat model is also controversially viewed as a suitable model for studying depressive behavior, anxiety, and exploratory dysfunction associated with PD [74,169,170,171,172,182,184,277,278]. To assess whether depression- and/or anxiety-like activities seem to be dependent on motor impairment, we performed a correlation analysis of drug-induced behaviors.

### 3.1. Readout of Test Results Indicating Anxiety-Like or Depression-Like Behavior

#### 3.1.1. Anxiety

In the OFT exploiting the natural aversion of rodents to exposed fields, the most relevant parameter indicating positive emotionality and reduced anxiety is the time spent in the center of the arena [225,228,229,230,279,280,281,282,283]. Correspondingly, in the EPM, an increase in relative time spent on open arms can be interpreted as inversely correlated with anxiety [226,284,285,286,287,288]. Increased defecation and increased grooming can also be interpreted as anxiogenic activities. Both tests are well-validated anxiety assays [289], and it is generally accepted that both OFT and EPM should be assessed together [290,291,292].

#### 3.1.2. Anxiety-Like Behavior Is Not Induced in Hemi-PD Rats and Not Altered by Additional Intrastriatal BoNT-A

Hemi-PD animals showed a decrease in locomotor activity in the OFT, indicating that DA depletion impairs the locomotor activity of rats [293]. These results corroborate the significantly decreased scores of spontaneous movements compared with Sham-lesioned animals [74,169,172,182,184,294,295,296]. The reduced locomotor activity in the OFT seemingly depends on altered motor skills, however, probably in part also on the motivation to explore a novel arena [169,180,297].

All other parameters examined in the OFT related to curiosity and exploration activity (center time, rearing time, grooming frequency, number of boli) did not differ significantly between all four groups, corresponding to comparable results of other researchers [74,170,182,295,296]. Eskow Jaunarajs et al., (2010) showed that although entries to center and vertical movements in the center were reduced in hemi-PD rats lesioned in the left MFB, time in the center was not changed [182]. Similarly, Zhang et al. [74] and Sun et al. [295] showed a reduction in motor activity in hemi-PD rats, but no significant difference between the groups examined in terms of rearing activity. O’Connor et al. [296] and Campos et al. [170] showed a reduction in motor activity in the OFT in hemi-PD rats after a unilateral MFB lesion and no significant changes between the test groups in the parameters investigated in connection with curiosity and exploratory activity.

Interestingly, most other studies described changes only in the locomotor activity or selected parameters in the OFT [74,169,171,172,184] (for details see Supplementary Materials Table S1).

Our present study showed the results of parameters related to curiosity and exploration activity (walking speed, center time, rearing time, grooming frequency, and number of boli). Our results in the EPM showed that the walking speed and the percentage of time spent in the open arms during a 5-min period did not differ significantly in the four groups. These results correspond to results from Delaville et al. (2012), which showed that after unilateral 6-OHDA injection into the right MFB, DA depletion did not induce any anxiety-like behavior: the number of entries, as well as the time spent in open arms, did not change compared with Sham-lesioned animals. These authors [184] also found that DA depletion is necessary, but not enough on its own to induce an anxious phenotype: A single depletion of DA, noradrenaline (NA), and/or serotonin (5-HT) did not change the time spent in the open arms or the number of entries into open arms in the EPM, whereas 5-HT or NA depletion combined with that of DA induced anxiety-like behavior. Carvalho et al. [169] also demonstrated no significant differences between the hemi-PD and Sham-injected groups regarding the ratio between time spent on the open arms and time spent on the closed arms. Interesting, only 2 studies [295,296] described anxiety-like behavior in Sprague-Dawley hemi-PD rats: Sun et al., 2015 showed that the percent time on open arms and open arms entries decreased; O’Connor et al., 2016 described that 6-OHDA-lesioned animals significantly spent less time in the open arm and ventured less open arms entries than Sham-injected or naïve rats.

Since intrastriatal BoNT-A injection in hemi-PD rats did not change the parameters related to curiosity and exploratory activity or fear, the BoNT-A-induced reduction in ACh release in the striatum does not appear to have any significant influence on this behavior.

Taking together the results of the present study and the literature, hemi-PD rats do not appear to be a suitable model for the investigation of anxiety-like behavior.

#### 3.1.3. Depression

In the FST evaluating depression-like behavior, the most important signs of “lowered mood” and “despair” are the immobility-associated parameters, i.e., latency, time, and frequency [74,234,235,298,299]. Most studies described three predominant behaviors in the modified FST: immobility, swimming, and climbing (struggling) time, or they analyzed and interpreted the duration of the immobility time only [298,299,300,301,302]. Immobility time was defined as a lack of motion of the whole body consisting of the small movements necessary to keep the animal’s head above the water [303,304,305]. Long immobility time, which reflects a state of helplessness/despair, was regarded as depression-like behavior [171,299]. Porsolt et al. [299] originally introduced immobility saying “immobility induced in these experiments reflects a state of lowered mood in the rat” and in his original version only the immobility time was measured. Also, studies of the antidepressant properties after unilateral 6-OHDA lesion into the MFB almost only evaluated the immobility time [74,172,182] and none of the other measurable parameters (Figure 3).

The FST is the most frequently used test to evaluate depression-like behaviors, including those related to PD [306,307,308,309]. This test is based on the adoption that, when placing an animal in a container filled with water, it will first make efforts to escape but eventually will exhibit immobility that may be considered to reflect a measure of behavioral despair [74,298,299].

The FST is a very popular model in animal research for a number of reasons because it involves the exposure of the animals to stress, which was shown to play a role in the tendency toward major depression [310,311,312] and also because pharmacological treatment with antidepressants prior to the test has been shown to reduce immobility in the FST [313,314,315].

Therefore, it is used as a screening assay for novel drugs with potential antidepressant properties [313,316,317,318]. The procedure is relatively easy to perform and its results are quickly analyzed. Moreover, its sensitivity to a broad range of antidepressant drugs makes it a suitable screening test is one of the most important features leading to its high predictive validity [298].

Depression appears in a combination of a short immobility latency, a long immobility time, and a high immobility frequency, while reduced depression is accompanied by a long immobility latency, a short immobility time, and a low immobility frequency.

Additionally, with respect to struggling, rats behaving in a depression-like manner seemingly have short struggling latency, a short struggling time, and a high struggling frequency. Reduced “despair” leads to the contrary observations of a longer struggling latency, a longer struggling time, and a lower struggling frequency. The swimming time is thought to be a parameter with a weaker interpretation possibility than immobility and struggling. As can be seen in Figure 3A,C,F, the times for swimming, struggling, and latency can be summed up to the duration of the 10-min FST experiment.

In the present study, hemi-PD rats demonstrated increased depression-like behaviors compared with Sham- or non-injected rats; this was seen in increased struggling frequency and increased immobility frequency (Figure 3D,G). Comparable with numerous observations in which no prolongation of the immobility time was found in rats with 6-OHDA lesions [74,171,172,182,295,319], but in contrast to the only report by Zhang et al. [319], who describe an increase of the immobility time, we could not observe any increase of the immobility time in hemi-PD rats compared with animals with sham surgery (Figure 3F).

#### 3.1.4. Depression-Like Behavior in Hemi-PD Rats Is Decreased by Intrastriatal BoNT-A

Intrastriatal BoNT-A injection ameliorated key signs of “decreased mood” and “despair” [234,235] in hemi-PD rats, as demonstrated by the significant improvement of six of the seven parameters that were measured in the FST: in hemi-PD rats, BoNT-A significantly reduced swimming time (Figure 3A), increased struggling time (Figure 3C), decreased struggling frequency (Figure 3D), increased immobility latency (Figure 3E), decreased immobility time (Figure 3F), and decreased immobility frequency (Figure 3G).

In PD, both characteristic motor symptoms and a variety of non-motor symptoms are found [1,2,3,4,15,16,17,18,19,20,61]. Depression is one of the most frequent non-motor symptoms of PD [45,46,47,48,50,51,52,53,55], however, the underlying mechanisms of the PD-associated depression have not been adequately clarified [320,321,322]. Seemingly, PD-associated depression is caused by multiple factors including genetic predisposition, biochemical disturbances, and psychological events [323,324]. The pathophysiology of depression in PD might also relate to changes in the dopaminergic, noradrenergic, serotonergic, and cholinergic systems [26,321,325,326].

Post mortem in vitro receptor autoradiography and in vivo PET studies in PD patients do not show any clear results concerning D_1_ receptor density in the CPu even though DA concentration is drastically reduced: striatal D_1_ receptor density is described as being unaltered [327,328], decreased [329] or increased [330]. However, an obvious increase of D_2_/D_3_ receptor density of about 25% was found in human PD patients [331,332]. Noradrenaline transmitter concentration is reduced in the CPu of PD, noradrenergic α_1_ and α_2_ receptor densities in the CPu of PD patients are not changed [333]. The density of serotonergic 5HT_2A_ receptors in the CPu of PD patients is not altered [334], although 5-HT levels and typical serotonin metabolites and transporters are significantly decreased to various extents in these patients [335]. A hypercholinism of the CPu in PD is constantly found [105,106,107,108]. Studies on muscarinic acetylcholine receptor (mAch) binding in the CPu of PD patients using post-mortem brain tissue [336,337,338] provided contradictory results: Lange et al. [337] postulate a decrease in M_1_ receptor density in the striatum, McOmish et al. [338] found no change. Unaltered M_2_ receptor densities in PD brain tissue compared with controls are communicated [337,338], contrasting to increased M_3_ receptor density in the CPu of PD patients [338]. Moreover, nicotinic acetylcholine receptor (nAch) binding in the CPu of PD patients is reduced in most reports: Aubert et al. [336], using in vitro receptor autoradiography, reported a decrease by 74%, and Meyer et al. [339], using PET analysis with 2-[^18^F]FA-85380, found a reduction of 20%.

Interestingly, roughly comparable results can be found in the DA-depleted CPu of hemi-PD rats. In hemi-PD rats, a slightly elevated D_1_ receptor density was found in the CPu early post-lesion that decreased with increasing post-lesion survival. Nearly normal values were reached 9 months post-lesion [340]. A consistent increase of 20–40% of the D_2_/D_3_ receptor density in the CPu is revealed in the CPu [341,342,343,344,345,346,347,348,349,350,351,352], also seen in our own studies [340]. The analysis of α_1_ receptors in the CPu of hemi-PD rats did not reveal any alterations [340], however, α_2_ receptor density significantly increased by up to about 30% in the DA-depleted CPu nine months post-lesion. In hemi-PD rats, striatal 5HT_2A_ receptor density was massively and constantly reduced by 50% [340].

In hemi-PD rats, all mAch receptor densities were mainly decreased ipsilateral to the lesion: M_1_ receptor density—9%, M_2_ receptor density—16%, M_3_ receptor density resembled vehicle-injected rats [353]. Hemi-PD rats showed a massive ipsilateral decrease in striatal nAch receptor density of about 50% [353].

We hypothesize that intrastriatal BoNT-A application in hemi-PD rats reduced depression-like behavior due to changes in the densities of transmitter receptors. The CPu of the DA-depleted and intrastriatally BoNT-A-injected rats indeed displayed changes in the direction of a normalization of the 6-OHDA-induced pathology. In hemi-PD rats, D_1_ receptor density in the CPu was left unaltered by BoNT-A. BoNT-A significantly reduced the pathologically increased D_2_/D_3_ receptor density. Alpha_1_ and α_2_ receptor densities in the CPu were found unaltered after BoNT-A, as was the 5HT_2A_ receptor density [340].

Concerning muscarinic receptor densities in the CPu, BoNT-A did not change M_1_ receptors. Both receptor densities of M_2_ (agonist binding) and M_2_ (antagonist binding) showed a BoNT-A-induced significant normalization of interhemispheric differences. M_3_ receptor and nAch receptor densities were nearly unaltered after additional BoNT-A [353].

Taking together the results of BoNT-A-induced changes in receptor binding in hemi-PD rats, it can be speculated that the partial normalization can be correlated with an improvement of depression-like behaviors. Interestingly, significant correlations were found between various receptor densities and apomorphine-induced rotation behavior [340,353].

#### 3.1.5. Tail Suspension Test

The TST has been used extensively as an acute test of antidepressant-like efficacy. Briefly, when rodents are suspended by their tails with no possibility of escape, they will eventually enter a state of learned helplessness in which a significant amount of time is spent hanging immobile [236]. The test appears to have good predictive validity for antidepressant drugs, in that this immobility is reduced following pre-treatment with standard antidepressant agents [236,237]. We found that rats in all the experimental groups scored 0: they showed no pathological immobility time in the TST over a period of 60 s.

#### 3.1.6. Correlating Parameters Indicative for Anxiety- and Depression-like Behaviors

We also analyzed whether there was a correlation between the expression of anxiety parameters (EPM: % time in open arms; OFT: center/total time) and the expression of depression parameters (struggling frequency, immobility frequency, struggling time, immobility time) between the 6-OHDA + Sham and Sham + Sham groups or between the 6-OHDA + Sham and 6-OHDA + BoNT groups.

**Figure 10:**
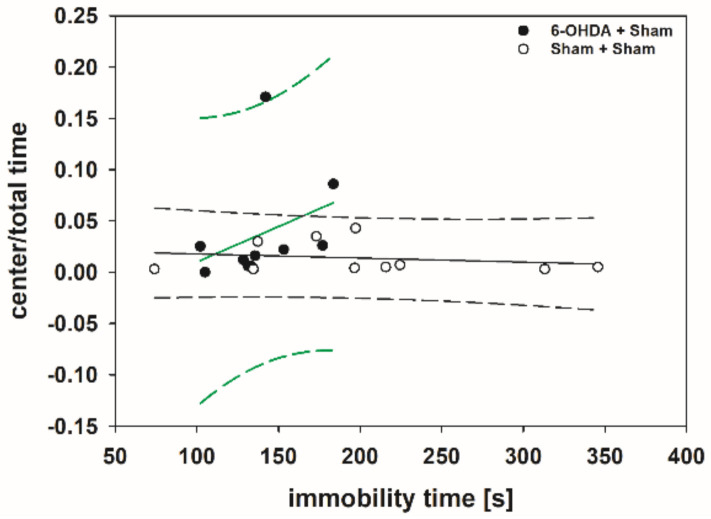
Scatter plot correlating the immobility time measured in the FST and center/total time measured in the OFT of rats of the 6-OHDA + Sham and Sham + Sham groups. Regression lines are displayed as solid lines and prediction intervals as dashed lines. Green-colored regression lines and prediction interval lines indicate significant correlation (*p* < 0.05).

There were no significant relationships between any pair of variables in the Spearman correlation table (*p* > 0.050) with only one exception (Supplementary Materials Table S2). The immobility time positively correlated with center/total time in the 6-OHDA + Sham group (rs = 0.620; *p* = 0.048) (Figure 10).

The positive correlation between the immobility time and center/total time in the 6-OHDA + Sham group means that, with increasing severity of the depression-like behavior, the severity of the anxiety-like behavior decreases. Although Ho et al. [354] found in naïve male Wistar rats no relationship between performance on the FST and EPM, a study from Estanislau et al. [355] in male Wistar rats reported an inverse relationship between performance on the FST and the EPM: animals that measured high on depression-like behaviors were low on anxiety-like behaviors [355,356]. Thus, this inverse relationship between depression- and anxiety-like behaviors shown in naïve animals corroborates our results in hemi-PD rats. Surprisingly, the results in rats diametrically oppose clinical observations in PD patients. Interestingly, depression in patients rarely strikes alone [354] in clinical populations, depression is highly comorbid with anxiety disorders with estimates as high as 80% [357].

#### 3.1.7. Correlating Open Field Test, Elevated Plus Maze Test, Forced Swim Test and Apomorphine- and Amphetamine-Induced Rotations

In PD, affective symptoms seemingly are not influenced by the severity of motor symptoms. Multiple groups have confirmed that neither depression nor anxiety are correlated with motor disability [49,358,359,360,361]. In fact, patients often report depression and anxiety although their motor status is improved by pharmacotherapy or neurosurgery [362,363,364].

In hemi-PD rats, correlation analysis was done to test if the outcome of the routinely used drug-induced rotation tests could possibly predict measures indicating depression-like or anxiety-like behavior in hemi-PD rats. Only a few significant correlations were found in rats of the 6-OHDA + Sham group. Increasing amphetamine-induced rotations correlated with decreasing anxiety-like behavior, as seen in the center/total time (%) in the OFT. Increasing apomorphine-induced rotations correlated with increasing depression-like behavior, as seen in the struggling latency in the FST.

It can be summarized that the parameters quantifying motor and non-motor behaviors are mostly not correlated in hemi-PD rats. Although an additional BoNT-A injection into the striatum of hemi-PD rats massively altered drug-induced rotation values and also improved depression-like behavior, respective significant correlations were not found in the 6-OHDA + BoNT group.

Thus, with respect to the missing correlation of parameters measuring depression or anxiety with motor disabilities, hemi-PD rats and PD patients are comparable.

## 4. Conclusions

In hemi-PD rats, intrastriatally applied BoNT-A has a positive effect on motor dysfunction without impairing cognitive and peripheral cholinergic functions [109,110,111,112,113,114,115,116,117,118,119,120].

The effects of BoNT-A injected into the dopamine-depleted CPu are reminiscent of the actions of several antidepressant agents [365,366]. Data suggest that BoNT-A is not only useful to improve motor behavior, but also could positively influence non-motor symptoms, such as depression and neurorestorative mechanisms in Parkinsonism.

Thus, locally applied BoNT-A, or other botulinum neurotoxins might be useful in treating brain dysfunctions requiring a deactivation of local brain activity, especially by lowering the availability of acetylcholine. Advantageously, the effect of local BoNT-A is time-limited and reversible. Optimistically, in the future and after experiments in primates, BoNTs might be used in clinical application as an effective and individually-tailored “chemical neurosurgical approach” as a local anticholinergic drug for treating striatal hypercholinism in PD and possibly also epilepsy [367,368].

## 5. Materials and Methods

### 5.1. Animals

Fifty-one young adult, 3-month-old, male Wistar rats (strain Crl:WI BR) weighing 250–290 g and obtained from Charles River Wiga (Sulzfeld, Germany) either assigned to non-injected (naïve controls, *n* = 12), controls (Sham 6-OHDA + Sham BoNT-A, further on named Sham + Sham, *n* = 11), Sham BoNT-A-injected hemi-PD rats (6-OHDA + Sham BoNT-A, further named 6-OHDA + Sham, *n* = 10), and BoNT-A-injected hemi-PD rats (further named 6-OHDA + BoNT, *n* = 18) were used for this study. Animals were housed in standard cages in a temperature-controlled room (22 °C ± 2 °C) under a 12 h light/dark cycle with free access to food and water 24 h a day. All procedures used in the present study complied with the guidelines on animal care. All experiments were approved by the State Animal Research Committee of Mecklenburg-Western Pomerania (LALLF M-V 7221.3-1.1-003/13, 26 April 2013; LALLF M-V 7221.3-1-056/18, 26 November 2018).

### 5.2. Injections of Drugs

#### 5.2.1. 6-OHDA Lesion Surgery

All surgical manipulations were carried out under aseptic conditions. Animals, weighing 290–310 g at the time of the first surgery, were deeply anesthetized by intraperitoneal (i. p.) injection of ketamine (50 mg kg^−1^ body weight (BW)) and xylazine (4 mg kg^−1^ BW).

**Figure 11:**
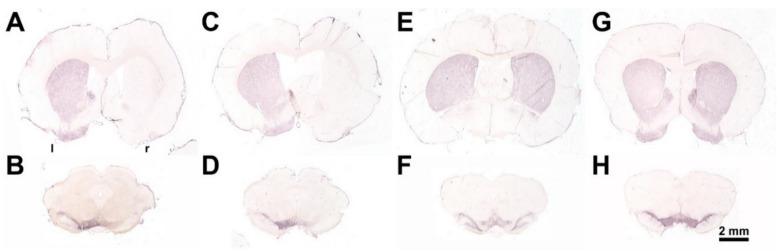
Representative frontal sections immunohistochemically stained against tyrosine hydroxylase showing the telencephalon (**A**,**C**,**E**,**G**) and the midbrain including substantia nigra pars compacta (**B**,**D**,**F**,**H**) from the experimental groups. (**A**,**B**) 6-OHDA + Sham BoNT-A; (**C**,**D**) 6-OHDA + BoNT-A; (**E**,**F**) Sham 6-OHDA + Sham BoNT-A; (**G**,**H**) non-injected. l, r: left and right hemispheres.

To induce an experimental hemi-PD, unilateral injection of 24 μg 6-hydroxydopamine hydrochloride (6-OHDA, Sigma-Aldrich, St. Louis, MO, USA) dissolved in 4 μL 0.1 M citrate buffer over 4 min via a 26-gauge 5 μL Hamilton syringe was performed into the right MFB using a stereotactic frame (Stoelting, Wood Dale, IL, USA). The Sham hemi-PD rats received 4 μL of the 0.1 M citrate buffer. Thereafter, the needle was left in place for a further 5 min to avoid reflow. The stereotactic injection was performed at the following coordinates with reference to bregma: anterior-posterior = −2.3 mm, lateral = 1.5 mm to the right, ventral = −9.0 mm [369]. The success of the lesion was verified by an apomorphine-induced rotation test [370,371] 4 weeks after the 6-OHDA application. Animals displaying more than four contralateral rotations/min—indicating a unilateral death of about 97% of the nigrostriatal DAergic neurons [371]—were tested further. Finally, brains were studied morphologically by TH-immunohistochemistry. 6-OHDA injected unilaterally into the right medial forebrain bundle (MFB) induced nearly complete death of dopaminergic neurons of the respective SNpc [109,110,118,120] and degeneration of nigrostriatal fibers and terminals in the striatum (Figure 11).

#### 5.2.2. Injection of BoNT-A into the Striatum

Six weeks after the 6-OHDA lesion, animals underwent a second stereotactic surgery. Under the same operative conditions, a solution of either BoNT-A (dissolved in phosphate-buffered saline supplemented with 0.1% bovine serum or vehicle (equal amount of PBS–BSA 0.1%) according to the same procedure (Sham BoNT-A) was injected into the right CPu at two sites [109,110,111,112,113,114,115,116,118,120]. Thus, rats received 2 × 1 μL BoNT-A solution (Lot No. 13028A1A; List, Campbell, CA, USA; purchased via Quadratech, Surrey, UK) containing a total of 1 ng BoNT-A (*n* = 18) or vehicle solution (*n* = 10). The respective coordinates with reference to bregma were: anterior-posterior = +1.3/−0.4 mm, lateral = 2.6/3.6 mm to the right, and ventral = −5.5 mm [369]. After each injection applied over 4 min, the cannula was left in place for another 5 min before being retracted to allow diffusion of the drug. BoNT-A was handled and stored according to the manufacturer’s instructions.

### 5.3. Behavioral Testing

Drug-induced rotational behavior, following apomorphine and amphetamine application, was measured about 4 weeks after the 6-OHDA or vehicle injection into the right MFB, and also 4 weeks after the ipsilateral intrastriatal injection of BoNT-A or vehicle, with the interval of 3 days between apomorphine and amphetamine application.

Spontaneous motor tests, i.e., open field, elevated plus maze, forced swim, and tail suspension tests were performed 4 weeks after the intrastriatal injection of BoNT-A or vehicle with the interval of 3 days between every single test.

One hour before starting each behavior test, the animals were kept in the examination room for about 1 h to become familiar with the novel environment.

#### 5.3.1. Drug-Induced Rotation Tests (Apomorphine, Amphetamine)

##### Apomorphine-Induced Rotation Test

To verify the success of the 6-OHDA lesion, the apomorphine-induced turning rate was determined 4 weeks after the 6-OHDA or vehicle injection. This test provides a sensitive and rapid behavioral correlate of the basal ganglia circuit disturbance caused by the unilateral lesion of the SNpc [370,372,373]. Apomorphine (0.25 mg/kg, Teclapharm, Lüneburg, Germany) dissolved in saline was injected i. p. and, after a waiting time of 5 min to ensure cerebral uptake, rotations were monitored for 40 min in a self-constructed, automated rotometer, modified according to Ungerstedt and Arbuthnott [371]. Full rotations of 360° were counted over 40 min and the mean number of rotations per minute was calculated (anti-clockwise: +, clockwise: −).

##### Amphetamine-Induced Rotation Test

Three days following the apomorphine testing, the amphetamine-induced rotational behavior was monitored as well [374,375,376], for the degree of rotational behavior after both drugs, apomorphine, and amphetamine, seemingly do not correlate in hemi-PD rats [376,377,378]. Rats were intraperitoneally injected with D-amphetamine sulfate (2.5 mg/kg, dissolved in 0.9% NaCl, Sigma Aldrich, München, Germany), and after a waiting time of 15 min were monitored for 60 min as described above.

#### 5.3.2. Spontaneous Motor Tests

##### Open Field Test

The spontaneous horizontal locomotor activity, anxiety, and willingness to explore a new environment were evaluated via the OFT, originally described by Hall [225,379]. Rats were adapted during the dark phase for 1 h before testing in the examination room. Animals were individually placed in a square OFT arena of 50 × 50 cm (walls 40 cam high), which was positioned inside an isolation box (TSE Systems, Bad Homburg, Germany). The 16 quadratic subfields of the square were divided into 12 peripheral and 4 central areas by a grid in the tracking software. Illumination of the OFT was provided by a white photo bulb at 450 l×. Animals were monitored online by a video camera placed inside the isolation box and tracked using the VideoMot2 Software (TSE Systems, Bad Homburg, Germany). This paradigm mimicked the natural conflict in rats between the tendency to explore a novel environment and the tendency to avoid a brightly lit open arena [380,381]. Rats were tested once for 10 min, then the apparatus was cleaned with 70% ethanol and dried with paper towels before testing the next animal to avoid odor interference in the test response. The walking speed of the animals, the time spent in the center and the edges of the OFT arena, the rearing time, and the grooming frequency [382,383] were evaluated.

##### Elevated Plus Maze Test

To assess the exploration, the locomotor activity, and anxiety-related behavior level, a self-made EPM was used [117]. This test is based on the natural aversion of rats for open and elevated areas [384]. The EPM apparatus consisted of two open arms and two closed arms (arm length 425 mm, arm width 145 mm, wall height 225 mm, width of ledges 10 mm) arranged in a way that two pairs of identical arms were placed opposite to each other. The arms emerged from a central platform and the entire apparatus was raised to a height of 90 cm above floor level. One h before the test start, the animals were kept in a dark phase in the examination room. Under dim light conditions (red photo bulb, 3.5 l×), rats were placed individually on the central platform facing one open arm. In between, the maze was carefully cleaned with a wet towel. During the 5 min test, anxiety was evaluated through two behavioral parameters: (1) time on the open arms (s), (2) presence on open arms (% open time), and (3) walking speed. All measures are inversely related to the anxiety level in rodents. Since anxiolytic and anxiogenic effects can be confounded by changes in motor activity, locomotion was additionally evaluated based on closed arm entries and total arm entries [284]. These parameters are considered to be the best indicators of the locomotor activity of rodents placed on the EPM [228]. The operational criterion for arm entry was the presence of the whole body and all four paws on the arm. Behavior was recorded with a video camera mounted directly 1 m above the EPM apparatus and tracked using the VideoMot2 Software (TSE Systems, Bad Homburg, Germany).

##### Forced Swim Test

The traditional FST was developed by Porsolt et al. [299] and is the most widely used and well-validated test for assessing depression-like behavior in rats. It has been successfully used to screen the efficacy of new antidepressant drugs [231,299,385,386] and has reasonably good predictive value for antidepressant potency in humans [387]. For our experiments, we used a modified, animal-friendly version of the forced swim test according to Gregus et al. [388].

The test was conducted in a Plexiglas swim tank (25 cm wide × 60 cm high) with no top. The tank was filled with 27 °C (±2 °C) water to a depth of 30 cm. Each rat was carefully placed into the forced-swim tank for 10 min. During this time, the rat’s behavior was recorded and monitored with a video camera mounted 1 m directly above the tank and tracked using the VideoMot2 Software (TSE Systems, Bad Homburg, Germany). At the end of the 10 min, the rat was removed from the tank, dried off with a towel, and placed back into its home cage to further dry under a heat lamp for 20 min. The water in the tank was changed after each rat. Behaviors scored in the test included: (1) time spent struggling, observed when the rat’s front paws break the surface of the water and the rat actively searches and struggle to get out of the tank; (2) time spent swimming, observed when the rat makes active swimming motions that are beyond those needed to simply stay afloat, but less than those observed when struggling; (3) time spent immobile, observed when the rat makes very few (enough to keep from drowning) movements with its body [389]. The latency to become immobile was scored as well. Depression-like behavior was inferred from increases in time spent immobile during the test [299].

##### Tail Suspension Test

The TST is a behavioral despair model of depression [236,390,391]. It is mostly applied in rodents to predict antidepressant potential by a decreasing immobility period produced by several different classes of antidepressant drugs [392,393]. TST is commonly used for mice and adapted to rats [394,395]. We utilized a modified version of the tail suspension test: rats were slowly lifted by their tails—grasping the base of the tail—for a total of 60 s. The time (s) the rat spent immobile, a correlate of depression-like behavior was analyzed.

### 5.4. Data Analysis

In general, an overall significance level = 0.05 was used. Normally distributed data were subjected to one-way ANOVA using SigmaPlot 14 Software (Systat Software, Inc., San Jose, CA 95110, USA). In the case of statistically significant different mean values, data were subjected to all pairwise multiple comparison procedures (Holm–Sidak method).

If the normality test (Shapiro–Wilk) or equal variance test (Brown–Forsythe) failed, a Kruskal–Wallis one-way ANOVA on ranks was done. In the case of statistically significant different median values among the treatment groups, a multiple comparison procedure (Dunn’s Method) was used.

To determine the strength of association of each behavioral test to apomorphine- or amphetamine-induced rotations, we performed Spearman rank-order correlation analyses. Spearman rank-order correlation is a nonparametric test that does not require the data points to be linearly related with a normal distribution about the regression line with constant variance. The Spearman rank-order correlation coefficient does not require the variables to be assigned as independent and dependent. Instead, only the strength of association is measured. Regression lines and prediction intervals were inserted into the resulting scatter plots. Prediction intervals also called the confidence interval for the population, describe the range where the data values will fall a percentage of the time for repeated measurements.

## Data Availability

Not applicable.

## Supplementary Materials

The following are available online at https://www.mdpi.com/article/10.3390/toxins13070505/s1, Table S1: Overview of the preclinical studies investigating the depressive- and anxiety-like behavior after unilateral lesion into the MFB in rats, Table S2: Spearman correlation table. Green colored *p* values indicate significant correlation.

## Abbreviations

α_1_	noradrenergic α_1_ receptor
α_2_	noradrenergic α_2_ receptor
ACC	anterior cingulate cortex
ACh	acetylcholine
BNST	bed nucleus of the stria terminalis
BoNT-A	botulinum neurotoxin-A
BSP	blepharospasm
CPu	caudate-putamen
DA	dopamine
D_1_	dopamine D_1_ receptor
D_2_	dopamine D_2_ receptor
D_3_	dopamine D_3_ receptor
dmPFC	dorsomedial prefrontal cortex
EPM	elevated plus maze test
FST	forced swim test
5-HT	serotonin
5HT_2A_	serotonergic 5HT_2A_ receptor
hemi-PD	hemiparkinsonian
HPC	hippocampus
iFC	intrinsic functional connectivity
mAch	muscarinic acetylcholine receptor
MAOI	monoamine-oxidase inhibitors
MAO-B	monoaminooxidase B
MDD	major depressive disorder
MFB	medial forebrain bundle
M_1_	muscarinic M_1_ receptor
M_2_	muscarinic M_2_ receptor
M_3_	muscarinic M_3_ receptor
NA	noradrenaline
nAch	nicotinic acetylcholine receptor
OFT	open field test
6-OHDA	6-hydroxydopamine
PD	Parkinson’s disease
PET	positron emission tomography
PFC	prefrontal cortex
rs	Spearman’s correlation coefficient
SNpc	substantia nigra pars compacta
SNRI	norepinephrine reuptake inhibitors
SSRI	selective serotonin reuptake inhibitors
TCA	tricyclic antidepressants
TST	tail suspension test
