# Supplementary material for: Antidepressant-Like Properties of Intrastriatal Botulinum Neurotoxin-A Injection in a Unilateral 6-OHDA Rat Model of Parkinson’s Disease
Source: Toxins (Basel). 2021 Jul 20;13(7):505. doi: 10.3390/toxins13070505 (PMC8310221; doi:10.3390/toxins13070505)
Supplement: Supplementary file 1 [file toxins-13-00505-s001.zip › toxins-1283773-supplementary.pdf]

Supplementary Materials: Antidepressant-Like Properties of Intrastriatal Botulinum Neurotoxin-A Injection in a Unilateral 6-OHDA Rat Model of Parkinson's Disease

Veronica Antipova, Carsten Holzmann, Alexander Hawlitschka, Martin Witt and Andreas Wree

Table S1. Overview of the preclinical studies investigating the depressive- and anxiety-like behavior after unilateral lesion into the MFB in rats.

Study	Rat Strain Male	Unilateral Injection into Medial Forebrain Bundle (MFB)	Depression/Test/Parameter/ Results	Anxiety/Test/Parameter/Results
Carvalho et al. 2013 [1]	Wistar-Han	Right MFB	**Sucrose preference test**: the preference for a sweet solution was shown to be significantly affected by unilateral 6-OHDA lesion of the MFB in 6-OHDA groups compare to Sham-lesioned group.	**OFT**: Total distance traveled and number of ambulatory episodes.<br><br>6-OHDA-lesioned group displaying a significantly lower motor activity when compare to Sham-operated rats (decreased total distance travelled) and overall reduction in movement initiation (decreased ambulatory episodes) caused by 6-OHDA lesion.<br><br>**EPM**: The ration between time in open arms and in closed arms was measured.<br>No significant differences were found between the 6-OHDA and Sham groups regarding the ratio between

				time spent on the open arm and time spent on closed arms.
Delaville et al. 2012 [2]	Wistar	Right MFB	**FST**: Immobility: 6-OHDA depletion alone did not produce any changes in "depressive-like" behavior compare to Sham-lesioned animals.<br><br>**Sucrose preference test**: 6-OHDA-lesioned rats did not show any changes in sucrose consumption compared to Sham-lesioned animas.	**OFT**: Locomotor activity.<br><br>6-OHDA-lesioned group displaying a significantly lower locomotor activity than Sham animals.<br><br>**EPM**: Time spent in the open and closed arms and number of entries into the open and closed arms.<br><br>No significant differences were found between the 6-OHDA and Sham groups regarding the number of entries, as well as the time spent in open arms.
Eskow Jaunarajs et al. 2010 [3]	Sprague-Dawley	Left MFB	**FST:** Immobility; Climbing; Swimming.<br><br>Immobility: no significant increase of immobility (no significant changes).<br><br>Climbing: a significant effect on climbing behavior (decrease).<br><br>Swimming: no significant changes.	**Locomotor chambes** (motor and anxiety-like behaviors): significantly reduced total distance traveled in hemi-PD group compare to Sham group by approx. 50%.<br><br>**Social interaction test**: reduction in frequency to approach the stimulus animal in DA-lesioned rats compare to Sham-lesioned group; significant decrease of anogenital sniffing, but not other sniffing, or flights.

			Unilateral DA lesion not alter depression-like behavior in DA-lesioned rats measured by the FST.	Unilateral DA lesion exerted anxiogenic effects on locomotor activity and social interaction.
Hui et al. 2014 [4]	Sprague-Dawley	Right MFB	**FST**: Immobility time: there was a strong tendency for 6-OHDA-lesioned rats to be more immobile than Sham-operated rats.<br><br>**Sucrose preference test**: rats showed the decreased sucrose consumption compared to Sham-operated rats.	**OFT**: Number of squares crossed (horizontal locomotion); number of rearings (vertical activity).<br><br>Unilateral 6-OHDA lesion decreased the number of squares crossed (horizontal movement) and rearing (vertical movement) - striking impairments in horizontal and vertical activity.
Liu et al. 2015 [5]	Wistar	Left MFB	**FST**: Immobility time: there was a strong tendency for 6-OHDA-lesioned rats to be more immobile than Sham-operated rats.<br><br>**Sucrose preference test**: unilateral 6-OHDA lesion of the MFB in rats significantly decreased sucrose consumption when compare to Sham-operated rats.	**OFT**: Number of squares crossed (horizontal locomotion); number of rearings (vertical activity).<br><br>Unilateral 6-OHDA lesion decreased the number of squares crossed (horizontal movement) and rearing (vertical movement).

O'Connor et al. 2016 [6]	Sprague-Dawley	Right MFB	Not done	**OFT**: The total distance traveled; time spent in center; the number of entries into the center and latency to enter the center.<br><br>6-OHDA-lesioned group displaying a significantly lower locomotor activity (distance traveled) than Sham animals, but no significant differences were found between the 6-OHDA-lesioned animals and Sham-lesioned animals in time in center; center crossing; latency to enter the center.<br><br>**EPM**: Time spent in the open arm and number of open and closed arm entries.<br><br>6-OHDA animals significantly spent less time in the open arm (open arms entries) than Sham rats or naïve rats. Also, 6-OHDA animals significantly spent less time in the closed arm (closed arms entries) than Sham rats but not naïve rats – implying that 6-OHDA animals have high baseline anxiety-like behavior and 6-OHDA lesioning is anxiogenic.
Sun et al. 2015 [7]	Sprague-Dawley	Right MFB	Not done	**OFT**: Number of squares crossed (horizontal locomotion); number of

				rearings (vertical activity); the percent of time spent in the central area.<br><br>Unilateral 6-OHDA lesion decreased the number of squares crossed (horizontal movement) and rearing (vertical movement), and decreased the percent of time spent in central area in the OFT.<br><br>**EPM**: The percent of time spent in open arms and the percent of entries in open arms.<br><br>Unilateral 6-OHDA lesion decreased the percent of open arm time and open arm entries.
Zhang et al. 2011 [8]	Sprague-Dawley	Right MFB	**FST**: Time immobile: a strong trend for unilaterally 6-OHDA-lesioned rats to be immobile compared to control group.	**OFT**: Horizontal activity: no significant differences between 6-OHDA and control group.<br><br>Rearing: no significant differences between 6-OHDA and control group.
Zhang et al. 2015 [9]	Sprague-Dawley	Right MFB	**FST**: Immobility time: unilateral lesioning MFB increased immobility time when compare to Sham-operated rats.<br>**Sucrose preference test**: rats showed the decreased	**OFT**: Number of squares crossed (horizontal locomotion); number of rearings (vertical activity).<br>Unilateral lesion decreased the number of squares crossed (horizontal movement) and rearing (vertical

	sucrose consumption compared to Sham-operated rats.	movement) - striking impairments in horizontal and vertical activity.

Table S2. Spearman correlation table.

Treatment Group	FST Parameter	Anxiety-Related Parameter	rs	*p*	Number of Samples
6-OHDA + BoNT	struggling frequency	EPM %time on open arms	−0.116	0.638	18
6-OHDA + Sham	struggling frequency	EPM %time on open arms	0.201	0.559	10
Sham + Sham	struggling frequency	EPM %time on open arms	−0.237	0.49	10
6-OHDA + BoNT	struggling time	EPM %time on open arms	−0.174	0.482	18
6-OHDA + Sham	struggling time	EPM %time on open arms	−0.0788	0.811	10
Sham + Sham	struggling time	EPM %time on open arms	−0.127	0.707	10
6-OHDA + BoNT	immobility frequency	EPM %time on open arms	−0.0836	0.736	18
6-OHDA + Sham	immobility frequency	EPM %time on open arms	0.360	0.292	10
Sham + Sham	immobility frequency	EPM %time on open arms	−0.079	0.811	10
6-OHDA + BoNT	immobility time	EPM %time on open arms	−0.0175	0.941	18
6-OHDA + Sham	immobility time	EPM %time on open arms	0.382	0.258	10
Sham + Sham	immobility time	EPM %time on open arms	0.309	0.365	10
6-OHDA + BoNT	struggling frequency	OFT center/total time	0.180	0.466	18
6-OHDA + Sham	struggling frequency	OFT center/total time	0.0306	0.919	10
Sham + Sham	struggling frequency	OFT center/total time	−0.00617	0.973	10
6-OHDA + BoNT	struggling time	OFT center/total time	−0.108	0.662	18
6-OHDA + Sham	struggling time	OFT center/total time	−0.419	0.213	10
Sham + Sham	struggling time	OFT center/total time	0.0308	0.919	10
6-OHDA + BoNT	immobility frequency	OFT center/total time	0.159	0.519	18
6-OHDA + Sham	immobility frequency	OFT center/total time	-0.107	0.759	10
Sham + Sham	immobility frequency	OFT center/total time	0.198	0.559	10
6-OHDA + BoNT	immobility time	OFT center/total time	0.138	0.574	18
6-OHDA + Sham	immobility time	OFT center/total time	0.620	0.048	10
Sham + Sham	immobility time	OFT center/total time	0.135	0.681	10

Green colored *p* values indicate significant correlation.

References

1. Carvalho, M. M.; Campos, F. L.; Coimbra, B.; Pêgo, J. M.; Rodrigues, C.; Lima, R.; Rodrigues, A. J.; Sousa, N.; Salgado, A. J. Behavioral characterization of the 6-hydroxidopamine model of Parkinson's disease and pharmacological rescuing of non-motor deficits. *Mol. Neurodegener.* **2013**, *8*, 14, doi:10.1186/1750-1326-8-14.
2. Delaville, C.; Chetrit, J.; Abdallah, K.; Morin, S.; Cardoit, L.; De Deurwaerdère, P.; Benazzouz, A. Emerging dysfunctions consequent to combined monoaminergic depletions in parkinsonism. *Neurobiol. Dis.* **2012**, *45*, 763–773, doi:10.1016/j.nbd.2011.10.023.
3. Eskow Jaunarajs, K. L.; Dupre, K. B.; Ostock, C. Y.; Button, T.; Deak, T.; Bishop, C. Behavioral and neurochemical effects of chronic L-DOPA treatment on nonmotor sequelae in the hemiparkinsonian rat. *Behav. Pharmacol.* **2010**, *21*, 627–637, doi:10.1097/FBP.0b013e32833e7e80.
4. Hui, Y. P.; Zhang, Q. J.; Zhang, L.; Chen, L.; Guo, Y.; Qiao, H. F.; Wang, Y.; Liu, J. Activation of prelimbic 5-HT1A receptors produces antidepressant-like effects in a unilateral rat model of Parkinson's disease. *Neuroscience* **2014**, *268*, 265–275, doi:10.1016/j.neuroscience.2014.03.033.
5. Liu, K. C.; Li, J. Y.; Tan, H. H.; Du, C. X.; Xie, W.; Zhang, Y. M.; Ma, W. L.; Zhang, L. Serotonin6 receptors in the dorsal hippocampus regulate depressive-like behaviors in unilateral 6-hydroxydopamine-lesioned Parkinson's rats. *Neuropharmacology* **2015**, *95*, 290–298, doi:10.1016/j.neuropharm.2015.03.031.
6. O'Connor, K. A.; Feustel, P. J.; Ramirez-Zamora, A.; Molho, E.; Pilitsis, J. G.; Shin, D. S. Investigation of diazepam efficacy on anxiety-like behavior in hemiparkinsonian rats. *Behav. Brain Res.* **2016**, *301*, 226–237, doi:10.1016/j.bbr.2015.12.045.
7. Sun, Y. N.; Wang, T.; Wang, Y.; Han, L. N.; Li, L. B.; Zhang, Y. M.; Liu, J. Activation of 5-HT1A receptors in the medial subdivision of the central nucleus of the amygdala produces anxiolytic effects in a rat model of Parkinson's disease. *Neuropharmacology* **2015**, *95*, 181–191, doi:10.1016/j.neuropharm.2015.03.007.
8. Zhang, X.; Egeland, M.; Svenningsson, P. Antidepressant-like properties of sarizotan in experimental Parkinsonism. *Psychopharmacology (Berl).* **2011**, *218*, 621–634, doi:10.1007/s00213-011-2356-7.
9. Zhang, Q. J.; Du, C. X.; Tan, H. H.; Zhang, L.; Li, L. B.; Zhang, J.; Niu, X. L.; Liu, J. Activation and blockade of serotonin7 receptors in the prelimbic cortex regulate depressive-like behaviors in a 6-hydroxydopamine-induced Parkinson's disease rat model. *Neuroscience* **2015**, *311*, 45–55, doi:10.1016/j.neuroscience.2015.10.016.
